# Serum osteoprotegerin and its gene polymorphisms in patients with Takayasu’s arteritis: a bicentric cross-sectional study

**DOI:** 10.1186/s42358-024-00384-w

**Published:** 2024-05-28

**Authors:** Camila da Silva Cendon Duran, Valéria de Falco Caparbo, Mittermayer Barreto Santiago, Bidossessi Wilfried Hounkpe, Ana Luisa Souza Pedreira, Isabella Vargas de Souza Lima, Henrique Ayres Mayrink Giardini, Virgínia Lucia Nazario Bonoldi, Diogo Souza Domiciano, Samuel Katsuyuki Shinjo, Rosa Maria R Pereira

**Affiliations:** 1 Division of Rheumatology, Faculdade de Medicina FMUSP, Universidade de Sao Paulo, Sao Paulo, SP Brazil; 2 Division of Rheumatology, Hospital Universitário Professor Edgard Santos, Universidade Federal da Bahia, Salvador, BA Brazil

**Keywords:** Genes, Osteoprotegerin, Polymorphisms, Takayasu’s arteritis, Vasculitis

## Abstract

**Introduction:**

Takayasu’s arteritis (TAK) patients are at an elevated risk of metabolic syndrome and cardiovascular diseases (CVD). Currently, there are no well-validated biomarkers to assess this risk in this population. Previous research in different cohorts has linked serum levels of osteoprotegerin (OPG) and its polymorphisms to accelerated atherosclerosis and a marker of poor prognosis in CVD. Thus, we assessed this protein as a potential biomarker of CVD in TAK patients.

**Objectives:**

To evaluate the serum levels of OPG and its SNPs (single nucleotide polymorphisms) in TAK patients and healthy controls, and to associate these parameters with clinical data.

**Methods:**

This bicentric cross-sectional study included TAK patients who were compared with healthy individuals (control group). The serum levels of OPG and the frequency of OPG SNPs [1181G > C (rs2073618), 245 A > C (rs3134069), 163T > C (rs3102735), and 209 C > T (rs3134070)] were compared between the both groups and associated with clinical data.

**Results:**

In total, 101 TAK patients and 93 controls were included in the study. The serum levels of OPG (3.8 ± 1.9 vs. 4.3 ± 1.8pmol/L, respectively; *P* = 0.059), and its four polymorphisms were comparable between both groups. In an additional analysis of only TAK patients, serum OPG levels and its four genes were not associated with any CVD parameters, except for higher OPG levels among patients without dyslipidemia.

**Conclusion:**

No significant differences were observed in serum OPG levels or in the genotype frequencies of OPG SNPs between the patient and control groups. Similarly, no correlation was found between laboratory parameters and clinical data on CVD risk in TAK patients.

## Introduction

Takayasu’s arteritis (TAK) is a chronic granulomatosis vasculitis that mainly affects large-caliber vessels and their major branches [1]. 

Cardiovascular (CV) involvement in TAK confers poor prognosis and can manifest as accelerated atherosclerosis, aneurysms, aortic dissection, pulmonary hypertension, coronary artery disease, valve abnormalities, and myocarditis [2]. Additionally, individuals with TAK had a high prevalence of metabolic syndrome and a higher Framingham score [3]. 

Osteoprotegerin (OPG), a glycoprotein of the tumor necrosis factor receptor superfamily, is expressed in osteoblasts, vascular smooth muscle cells, and endothelial cells. It is considered a marker of poor prognosis in CV disease, and its pathogenic role has been attributed to this glycoprotein [4]. Increased serum OPG levels have been reported in individuals with coronary artery disease, abdominal aortic aneurysms [5, 6], and metabolic syndrome [7]. Moreover, OPG polymorphisms such as G1181C and T950C are associated with coronary artery disease [6]. 

Patients with autoimmune rheumatic diseases have elevated CV diseases and risk factors. In this context, OPG levels are correlated with worse CV outcomes in patients with rheumatoid arthritis [8], systemic sclerosis [9], and systemic lupus erythematosus [10, 11]. Breland et al. [12] also observed increased OPG levels in patients with immune-mediated rheumatic and coronary artery disease.

To the best of our knowledge, only one study has analyzed serum OPG levels in a few samples of patients with TAK and found values similar to those of a control group [13]. As a limitation, this study did not assess serum OPG levels or its polymorphisms in relation to CV diseases and their risk factors, which are commonly observed in patients with TAK.

Therefore, the aim of the present study was to evaluate serum levels of OPG and its polymorphisms in a representative sample of patients with TAK from two Brazilian tertiary centers. Additionally, we analyzed the association between OPG and its polymorphisms and clinical and CV diseases in patients with TAK.

## Patients and methods

### Study design

This was a cross-sectional, bicentric study, in which patients with TAK and healthy individuals were included between January 2022 and October 2023.

The study was approved by the Ethics Committee (CAAE 53365521.9.0000.0068/53365521.9.3002.0049), and all patients signed an informed consent form.

### Patients and healthy individuals

All patients with TAK met the American College of Rheumatology / European League Against Rheumatism (ACR/EULAR) 2022 classification criteria [14]. Patients aged ≥ 45 years were excluded from the study.

Healthy individuals without TAK in an age group similar to that of patients with TAK were included in the control group.

### Patient data

In addition to the interviews, the following data were collected through analysis of patients’ medical records:

- Demographic and anthropometric data: age, sex, ethnicity, and body mass index (BMI);
- Clinical data: age at diagnosis, duration of disease, CV diseases, and their risk factors (hypertension, diabetes mellitus, dyslipidemia, smoking, family history of CV disease, first-degree relative with myocardial infarction, or stroke before the age of 55 and 65 years in men and women, respectively);
- Image rating: Hata angiographic classification [15] and aneurysm presence or absence;
- Disease activity assessment using the 2010 Indian Takayasu Clinical Activity Score (ITAS) - Brazilian Portuguese Version [16, 17]. The index was adapted to provide an extra score in cases of high acute phase reactants (erythrocyte sedimentation rate [ESR], reference value < 15 mm/1st hour), and C-reactive protein [CRP], reference value < 5 mg/dL) - ITAS2010-A. Disease activity was considered for a value of ≥ 2 for ITAS2010 and ≥ 5 for ITAS2010-A;
- Current treatment: use of glucocorticoids, immunosuppressive drugs, anticoagulants, antiplatelet agents, and cholesterol-lowering drugs.

### Laboratory analysis

Peripheral venous blood was collected from the laboratory to analyze the serum levels of OPG. Serum extracted from the subjects was stored at − 80 °C.

OPG was measured in duplicate using an enzyme-linked immunosorbent assay (ELISA, Biomedica, Vienna, Austria), according to the manufacturer’s instructions. The inter- and intra-assay coefficients of variation for OPG were 6.0% and 3.3%, respectively.

Genomic DNA was extracted from peripheral blood leukocytes using a Qiagen kit (QIAGEN GmbH, Hilden, Germany) for DNA extraction. After extraction, the samples were stored at − 20 °C. The SNPs in the OPG gene (8q24) [1181 G > C (rs2073618), 245 A > C (rs3134069), 163 T > C (rs3102735), and 209 C > T (rs3134070)] were analyzed. SNP genotyping assays were performed using the Taqman system (Applied Biosystems, Foster City, CA) and the StepOne Plus equipment (Applied Biosystems, Foster City, CA). To verify the quality of genotyping, 30 random samples were analyzed twice.

### Statistical analysis

Convenience sampling was performed. The Shapiro-Wilk test was used to verify the normality of the variables, and data were presented as mean ± standard deviation, median (interquartile 25th − 75th ), and frequency (%). Comparisons between patients and controls were performed using the Student’s *t*-test, Mann-Whitney U test, and chi-square test. Hardy-Weinberg equilibrium was assessed in patients and controls by comparing the observed SNP distribution with the expected distribution using the chi-square test implemented in the HardyWeinberg package in R (R CORE TEAM, 2013). Polymorphism frequencies were compared between groups with chi-square test using the SNPassoc package. Statistical significance was defined as *P* < 0.05.

## Results

### Patients with TAK vs. control group

A total of 101 and 93 individuals with TAK and controls, respectively, were included in this study. Current age (35.0 ± 7.5 vs. 35.0 ± 5.3 years), female sex distribution (83% vs. 84%), and BMI values (26.0 ± 5.6 vs. 26.0 ± 4.4 kg/m^2^) were comparable between groups (all *P* > 0.05).

The serum levels of OPG were similar between patients with TAK and controls (3.8 ± 1.9 vs. 4.3 ± 1.8 pmol/L, *P* = 0.059) (Table 1).

**Table 1:** Serum levels of osteoprotegerin, and its SNP genotype frequencies in patients with Takayasu’s arteritis and control group

	Genotype	TAK (*n* = 101)	Control (*n* = 93)	*P*
OPG (pmol/L)	-	3.8 ± 1.9	4.3 ± 1.8	0.059
OPG 1181 G > C	G/G G/C C/C	35 (34.7) 51 (50.5) 15 (14.9)	39 (41.9) 37 (39.8) 17 (18.3)	0.325
OPG 245 A > C	A/A A/C C/C	77 (76.2) 23 (22.8) 1 (1.0)	76 (81.7) 16 (17.2) 1 (1.1)	0.625
OPG 163 T > C	T/T C/T C/C	63 (62.4) 34 (33.7) 4 (4.0)	61 (65.6) 29 (31.2) 3 (3.2)	0.886
OPG 209 C > T	C/C C/T T/T	75 (74.3) 25 (24.8) 1 (1.0)	75 (80.6) 17 (18.3) 1 (1.1)	0.548

Data are expressed as mean ± standard, or deviation of frequency (%)

*OPG* osteoprotegerin; *TAK* Takayasu’s arteritis

The OPG allelic frequency distributions in the present sample (TAK and control groups) showed Hardy-Weinberg equilibrium for all analyzed polymorphism: rs2073618 (χ^2^ = 0.45, *P* = 0.50), rs3134069 (χ^2^ = 0.078, *P* = 0.78), rs3102735 (χ^2^ = 0.08, *P* = 0.77), rs3134070 (χ^2^ = 0.25, *P* = 0.62). Moreover, the genotype frequencies of the four OPG SNPs were comparable between the groups (Table 1).

### Patients with TAK

The mean age at the TAK diagnosis was 24.2 ± 9.1 years, with a mean disease duration of 11.0 ± 5.0 years, and 63.4% patients were White (Table 2). The Hata V angiographic classification was the most prevalent (66.3%), and 26 (25.3%) patients had aneurysms.

**Table 2:** Demographic, disease features, disease status, comorbidity, treatment, and lifestyle characteristics of patients with Takayasu’s arteritis

	TAK (*n* = 101)
Age at disease diagnosis (years)	24.2 ± 9.1
Disease duration (years)	11.0 ± 5.0
White ethnicity	64 (63.4)
Hata angiographic classification	
I	7 (6.9)
IIa	5 (5.0)
IIb	10 (9.9)
III	4 (4.0)
IV	8 (7.9)
V	67 (66.3)
Aneurysm	26 (25.7)
Disease activity (ITAS 2010-A)	17 (16.8)
Treatment	
Prednisone	
Current using	33 (32.7)
Current dose (mg/day)	10 (2.5–60.0)
Immunosuppressive agents	62 (61.3)
Anticoagulation	10 (9.9)
Antiplatelet agents	77 (76.2)
Statins	50 (50.0)
Hypertension	72 (71.3)
Dyslipidemia	54 (53.5)
Renal chronic disease	8 (8.0)
Acute myocardial infarction	7 (6.9)
Diabetes mellitus	5 (5.0)
Smoking	
Previous smoking	16 (15.8)
Current smoking	4 (4.0)
Family history of CV diseases	10 (9.9)

Data are expressed as mean ± standard, median (25th − 75th ), or frequency (%)

*CV* cardiovascular; *ITAS* Indian Takayasu Clinical Activity Score; *TAK* Takayasu’s arteritis

Seventeen (16.8%) patients had active disease according to ITAS2010-A, 33 (32.7%) were using prednisone, and 62 (61.3%) were using other immunosuppressive agents (Table 2).

Regarding CV diseases and their risk factors, 71.3% patients had hypertension, 53.5% had dyslipidemia, 8% had renal disease, 6.9% had acute myocardial infarction, and 5% had diabetes mellitus. A family history of CV disease was present in 9.9% of the patients.

A total of 15.8% of the patients had a history of smoking and 4% were current smokers.

Serum OPG levels were not associated with disease activity [(3.4 (2.5–3.5) vs. 3.6 (2.6–4.6) pmol/L, *P* = 0.676)], and treatment with glucocorticoids [(3.5 (2.5–4.6) vs. 3.4 (2.6–4.8) pmol/L, *P* = 0.806)] or immunosuppressive agents [(3.4 (2.4–4.6) vs. 3.7 (2.7–4.9) pmol/L, *P* = 0.567)]. OPG was not associated with any CV diseases or their risk factors, except for lower OPG levels in those with dyslipidemia than in those without dyslipidemia (Table 3).

**Table 3:** Median serum osteoprotegerin levels according to the presence of not or the disease activity and comorbidities in patients with Takayasu’s arteritis

Parameters	Present	Absent	*P*
Disease activity	3.4 (2.5–3.5)	3.6 (2.6–4.6)	0.676
Hypertension	3.4 (0.2–8.9)	4.0 (1.3–7.2)	0.447
Diabetes mellitus	2.3 (0.71–8.9)	3.6 (0.18–8.3)	0.168
Dyslipidemia	3.1 (0.2–8.9)	3.9 (0.3–8.3)	0.039
Renal chronic disease	3.3 (1.2–6.2)	3.7 (0.2–8.9)	0.669
Aneurysm	4.2 (2.8–5.7)	3.4 (2.4–4.5)	0.115
Myocardial infarction	3.2 (1.2–4.4)	3.6 (2.6–4.8)	0.186
Glucocorticoids	3.5 (2.5–4.6)	3.4 (2.6–4.8)	0.806
IS agents	3.4 (2.4–4.6)	3.7 (2.7–4.9)	0.567

Data are expressed as median (25th − 75th )

*IS* immunosuppressive

The four OPG SNPs distributions were also comparable in patients with TAK with respect to disease activity and comorbidities (Table 4).

**Table 4:** SNP genotype frequencies in patients with Takayasu’s arteritis, according to disease activity and comorbidities

Parameters	Polymorphism	Genotype	Presence	Absent	*P*
Disease activity	OPG 1181 G > C	G/G G/C C/C	9 (52.9) 6 (35.3) 2 (11.8)	25 (30.5) 44 (53.7) 13 (15.9)	0.219
	OPG 245 A > C	A/A A/C C/C	14 (82.4) 3 (17.6) 0	62 (75.6) 19 (23.2) 1 (1.2)	0.797
	OPG 163 T > C	T/T C/T C/C	11 (64.7) 6 (35.3) 0	52 (63.4) 26 (31.7) 4 (4.9)	< 0.999
	OPG 209 C > T	C/C C/T T/T	13 (76.5) 4 (23.5) 0	61 (74.4) 20 (24.4) 1 (1.2)	< 0.999
Hypertension	OPG 1181 G > C	G/G G/C C/C	24 (33.3) 39 (54.2) 9 (12.5)	11 (39.3) 11 (39.3) 6 (21.4)	0.343
	OPG 245 A > C	A/A A/C C/C	54 (75.0) 17 (23.6) 1 (1.4)	23 (82.1) 5 (17.9) 0	0.711
	OPG 163 T > C	T/T C/T C/C	43 (59.7) 25 (34.7) 4 (5.6)	20 (71.4) 8 (28.6) 0	0.408
	OPG 209 C > T	C/C C/T T/T	52 (72.2) 19 (26.4) 1 (1.4)	23 (82.1) 5 (17.9) 0	0.597
Diabetes mellitus	OPG 1181 G > C	G/G G/C C/C	0 4 (80.0) 1 (20.0)	35 (36.5) 47 (49.0) 4 (14.6)	0.223
	OPG 245 A > C	A/A A/C C/C	3 (60.0) 2 (40.0) 0	74 (77.1) 21 (21.9) 1 (1.0)	0.357
	OPG 163 T > C	T/T C/T C/C	3 (60.0) 1 (20.0) 1 (20.0)	60 (62.5) 33 (34.4) 3 (3.1)	0.339
	OPG 209 C > T	C/C C/T T/T	2 (40.0) 3 (60.0) 0	73 (76.0) 22 (22.9) 1 (1.0)	0.142
Dyslipidemia	OPG 1181 G > C	G/G G/C C/C	16 (29.6) 30 (55.6) 8 (14.8)	19 (40.4) 21 (44.7) 7 (14.9)	0.487
	OPG 245 A > C	A/A A/C C/C	42 (77.8) 12 (22.2) 0	35 (74.5) 11 (23.4) 1 (2.1)	0.806
	OPG 163 T > C	T/T C/T C/C	35 (64.8) 17 (31.5) 2 (3.7)	28 (59.6) 17 (36.2) 2 (4.3)	0.863
	OPG 209 C > T	C/C C/T T/T	41 (75.9) 13 (24.1) 0	34 (72.3) 12 (25.5) 1 (2.1)	0.727
Chronic renal disease	OPG 1181 G > C	G/G G/C C/C	5 (62.5) 3 (37.5) 0	30 (32.3) 48 (51.6) 15 (16.1)	0.227
	OPG 245 A > C	A/A A/C C/C	5 (62.5) 3 (37.5) 0	72 (77.4) 20 (21.5) 1 (1.1)	0.429
	OPG 163 T > C	T/T C/T C/C	4 (50.0) 4 (50.0) 0	59 (63.4) 30 (32.3) 4 (4.3)	0.603
	OPG 209 C > T	C/C C/T T/T	4 (50.0) 4 (50.0) 0	71 (76.3) 21 (22.6) 1 (1.1)	0.175
Aneurysm	OPG 1181 G > C	G/G G/C C/C	12 (46.2) 12 (46.2) 2 (7.7)	23 (30.7) 39 (52.0) 13 (17.3)	0.249
	OPG 245 A > C	A/A A/C C/C	18 (69.2) 8 (30.8) 0	59 (78.7) 15 (20.0) 1 (1.3)	0.417
	OPG 163 T > C	T/T C/T C/C	16 (61.5) 10 (38.5) 0	47 (62.7) 24 (32.0) 4 (5.3)	0.656
	OPG 209 C > T	C/C C/T T/T	17 (65.4) 9 (34.6) 0	58 (77.3) 16 (21.3) 1 (1.3)	0.404
Myocardial infarction	OPG 1181 G > C	G/G G/C C/C	2 (28.6) 4 (57.1) 1 (14.3)	33 (35.1) 47 (50.0) 14 (14.9)	0.927
	OPG 245 A > C	A/A A/C C/C	6 (85.7) 1 (14.3) 0	71 (75.5) 22 (23.4) 1 (1.1)	< 0.999
	OPG 163 T > C	T/T C/T C/C	5 (71.4) 2 (28.6) 0	58 (68.7) 32 (34.0) 4 (4.3)	< 0.999
	OPG 209 C > T	C/C C/T T/T	5 (71.4) 2 (28.6) 0	70 (74.5) 23 (24.5) 1 (1.1)	< 0.999

Data were expressed as frequency (%)

*OPG* osteoprotegerin

The frequency of each OPG SNPs did not differ between both Brazilian centers (*P* > 0.05).

## Discussion

This is the first study to assess the OPG SNP genotype frequencies in patients with TAK. This patient group is considered a high-risk population for CV disease and metabolic syndrome [3]. Our analysis did not identify a higher frequency of OPG SNPs when comparing patients with TAK and controls. When evaluating the circulating OPG levels in patients with TAK and controls, we found no differences between the two groups.

Our study has the advantage of being bicentric, which allowed us to evaluate a significant number of patients with this rare vasculitis. We also ensured that the control and patient groups were age matched, preventing this bias in data interpretation. This retrospective study model is a limitation. However, we limited the inclusion to patients who met the updated classification criteria (ACR/EULAR 2022) [14]. In addition, we excluded participants aged ≥ 45 years, considering that individuals in this age group could have age-related cardiovascular risk factors.

We also did not identify a difference in the frequency of OPG SNPs when evaluating patients with TAK associated with aneurysms, disease activity, or other comorbidities, such as hypertension, diabetes mellitus, smoking, dyslipidemia, history of myocardial infarction, or chronic renal disease. No association was found between serum levels of OPG and patients with TAK and other comorbidities.

Previous studies have associated circulating OPG levels with CV diseases and risk factors, such as age, smoking, hypertension, insulin resistance, obesity, diabetes mellitus, and chronic kidney disease [18, 19]. Serum OPG levels have also been used as biomarkers for the diagnosis and evaluation of abdominal aortic aneurysm growth [5, 20]. 

We evaluated a genetic marker that might be more prevalent in patients with TAK, particularly those with aneurysms, active disease, or a history of acute myocardial infarction. Additionally, we evaluated a potential biomarker capable of predicting CV disease risk in these patients with a higher mortality rate related to CV diseases due to chronic inflammatory process [21]. 

The physiopathology of the OPG/RANK/RANKL system in CV diseases and atherosclerosis is complex and not yet fully understood, and the role of OPG in this pathophysiology remains controversial. Animal studies have suggested a protective role for OPG in the formation of arterial calcification and its contribution to plaque stabilization, as demonstrated by OPG-deficient mice showing increased vascular calcification [22]. However, studies in humans suggest that OPG is related to greater development of peripheral arterial disease, coronary and cerebrovascular atherosclerosis, endothelial damage, vascular calcification, and aortic aneurysm [5, 20, 23]. These divergent results seem to be related to the stage of atherosclerotic lesions. In the early stages, OPG may activate inflammatory pathways and increase to compensate for the vascular damage. As the injury progresses, OPG may become harmful to vessels or fail to reverse vascular calcification [9]. 

## Conclusions

Based on our findings, OPG did not prove to be a marker for CV evaluation and prognosis in patients with TAK. More studies are needed to improve our understanding of the role of OPG in vascular calcification, aneurysm formation, and CV diseases. The search for biomarkers of cardiac risk in patients with rheumatic diseases is a reality and must be continued to prevent adverse outcomes.

## Data Availability

Not applicable.

## Abbreviations

ACR: American College of Rheumatology
BMI: Body Mass Index
CRP: C-Reactive Protein
CV: Cardiovascular
CVD: Cardiovascular Diseases
DNA: Deoxyribonucleic Acid
ESR: Erythrocyte Sedimentation Rate
EULAR: European League Against Rheumatism
ITAS: Indian Takayasu Clinical Activity Score
OPG: Osteoprotegerin
RANK: Receptor activator of nuclear factor κ B
RANKL: Receptor activator of nuclear factor kappa-Β ligand
SNP: Single nucleotide polymorphisms
TAK: Takayasu’s arteritis
